# Patterns of wildlife-vehicle collisions in Poland: a cross-taxonomic analysis of a nationwide citizen science dataset

**DOI:** 10.1038/s41598-026-52546-z

**Published:** 2026-05-12

**Authors:** Andrzej Wuczyński, Karol Kustusch, Przemysław Stolarz, Maciej Wuczyński, Clara Grilo, Tomé Neves, Federico Morelli

**Affiliations:** 1 Institute of Nature Conservation, Polish Academy of Sciences, Al. Mickiewicza 33, Kraków, 31-120 Poland; 2 Polish Roadkill Observation System, Waryńskiego 153, Grudziądz, 86-300 Poland; 3 Medical University of Warsaw, Banacha 1a, Warszawa, 02-097 Poland; 4 Statistical Analysis Centre, Wroclaw Medical University, Wrocław, 50-372 Poland; 5 CIBIO, Centro de Investigação em Biodiversidade e Recursos Genéticos, InBIO Laboratório Associado, Universidade do Porto, Campus de Vairão, Vairão, 4485-661 Portugal; 6 CIBIO, Centro de Investigação em Biodiversidade e Recursos Genéticos, InBIO Laboratório Associado, Instituto Superior de Agronomia, CIBIO, Universidade de Lisboa, Lisboa, 1349-017 Portugal; 7 BIOPOLIS Program in Genomics, Biodiversity and Land Planning, CIBIO, Campus de Vairão, Vairão, 4485-661 Portugal; 8 Department of Life Sciences and Systems Biology, University of Turin, Via Accademia Albertina 13, Turin, 10123 Italy

**Keywords:** Road ecology, Animal mortality, Traffic impact, Vertebrates, Temporal patterns, Citizen science, Ecology, Ecology, Zoology

## Abstract

Wildlife-vehicle collisions (WVCs) are a global biodiversity threat impacting most terrestrial vertebrates and posing scientific, economic and conservation challenges. Research on WVC is nevertheless geographically uneven, and large-scale multi-taxon studies are scarce. We analysed data from the Polish Roadkill Observation System, a nationwide volunteer-based programme recording WVC carcasses across all terrestrial vertebrates. Based on 28,709 casualties recorded during 2000–2022, we examined the mortality patterns in vertebrate classes and some correlates of WVCs distribution. In total, 205 species were identified, representing 55% of Poland’s terrestrial vertebrate fauna, including 16% of threatened species. The approximate proportion of roadkilled mammals, amphibians, birds and reptiles was 8:8:5:1; mammals and amphibians are thus the most frequently reported vertebrates on Polish roads by citizen scientists. Reports of multiple casualties concerned mostly amphibians and reptiles, and were often associated with protected areas. Regression models revealed that observed roadkill was positively correlated with body mass and carcass persistence but not with species distribution range in Poland. Prominent seasonal patterns were also evident, with peak mortality occurring in warm seasons and coinciding with taxon-specific phenological events. Our study demonstrates the value of citizen-science data for identifying nationwide patterns of WVCs, while also highlighting the need to move beyond opportunistic observations toward a nationally coordinated, systematic survey to generate more accurate and policy-relevant estimates.

**Supplementary Information:**

The online version contains supplementary material available at 10.1038/s41598-026-52546-z.

## Introduction

Although wildlife-vehicle collisions (WVCs) are a relatively new anthropogenic agent of animal mortality, their importance is already well-known because of their ever more serious economic consequences and their pervasive effect on biodiversity^1^. The latter is reflected in existing mortality estimates, e.g. 194 million birds were killed on roads annually in Europe^2^, 89–340 million in the USA^3^ and over 13 million in Canada^4^, 32 million vertebrate WVCs per year in Germany and 4 million in Belgium^5^(see Schwartz et al.^6^ for further estimates). These data clearly show that WVCs are a serious conservation problem that can significantly add to an array of other human-related and natural factors affecting wildlife^7–9^. Despite the global dimensions and general acknowledgement of WVCs, many regions and countries, such as Poland, do not recognize the magnitude and characteristics of the problem.

Species vulnerability to road traffic is influenced by life-history traits, habitat characteristics, and features of the transport network^10–13^. Small-bodied taxa, particularly herpetofauna, frequently account for a large proportion of WVCs^14,15^. Among these, amphibians experience some of the highest road mortality rates reported, often exceeding 10,000 individuals km⁻¹ year⁻¹^16^. Furthermore, amphibian road mortality is typically highly aggregated in both space and time, with mortality events concentrated at specific road sections and during short periods^17–19^. However, the relative contributions of different vertebrate groups are less clear when assessments are conducted at broader spatiotemporal scales. Widespread and year-round active animals, such as mammals and birds, may dominate WVC patterns at the scale of a large country or over multiple years, but their relative contributions will again depend on a range of factors, including taxa specific ecological properties, susceptibility to collisions, and sampling error associated with different carcass persistence and detection^20–22^.

WVCs frequency typically exhibits seasonal variations, but there is no consistency in the literature with regards to either the period of heightened mortality among taxonomic groups or the number of mortality peaks. Indeed, temporal patterns show a wide variation between vertebrate classes^11,15,23^ and species within classes^24–28^, between geographical locations and even nearby regions and roads^29,30^. For example, Erritzoe et al.^31^ compared the distribution of traffic mortality in various bird species between four geographically close European countries, but obtained quite different patterns in each one, reflecting geographic variation in species biology, such as breeding phenology, terms of fledging, and migration patterns. This heterogeneity significantly prevents the implementation of effective mitigation measures, so well-tailored data are essential^10,30^.

From the conservation perspective, the identification of roads with excessively high mortality and the presence of endangered species among WVCs are of special concern^16,32^, indicating areas of increased biodiversity and hence the need for mitigation efforts^33^. Any solution to these problems requires sufficiently large, flexible and recent datasets e.g.^34^. The rapid increase in citizen science programmes can meet these requirements. A number of open-access reporting systems launched worldwide have produced datasets quantifying biodiversity^35^ and an array of other disciplines, including WVCs^6,36–39^. However, their coverage is still insufficient and the problem of WVCs remains poorly recognized in most parts of the world, including biodiversity hotspots.

This study provides the first WVCs data in Poland, based on a citizen science project – the *Polish Roadkill Observation System* http://zwierzetanadrodze.pl, the only nationwide programme in Poland covering the complete set of terrestrial vertebrate species. The impact of traffic on wildlife mortality is an evident problem in Poland, but it is poorly understood. There are several papers examining the subject at a local scale^32,40–46^, but just a few studies have done so at the national scale^47–49^. The latter are, however, based on police records, which supply data only on collisions between vehicles and medium-sized and large mammals, thus a very limited range of vertebrates occurring in Poland.

Our objective was to perform the first synthesis of road mortality patterns for all classes of terrestrial vertebrates in Poland. We adopted a cross-taxonomic approach to show which vertebrate classes and which species in these classes most frequently perished on Polish roads according to citizen science data. We collated animal fatalities with nationwide species richness and conservation status, and analysed variables affecting WVCs distribution, such as the season, dominant environment, the species’ body mass and distribution range in Poland. We also aimed to highlight the value of a large volunteer-based database for detecting the broad patterns of road mortality and developing conservation strategies.

## Methods

### Study area

The data were collected all over Poland, a large Central European country with an area of 313,933 km^2^ (the ninth largest country in Europe) and a human population of over 38 million. Poland is a predominantly lowland country, but with mountains (max. alt. 2,503 m a.s.l.) and adjoining uplands in the south. The drainage system is relatively uniform, and the climate is mostly temperate with an average annual precipitation of ca. 600 mm. The dominant land cover types include farmland (61.5%), forest (30.2%), water (2.1%) and built-up areas (1.7%) (https://stat.gov.pl). Poland is known for its internationally important biodiversity^50^, with 32.6% of land being protected and with populations of charismatic and rare species, such as European Brown Bear (*Ursus arctos*), Wolf (*Canis lupus*), European Bison (*Bison bonasus*) and White Stork (*Ciconia ciconia*). These characteristics result from the extent of the country, the very large areas of traditional farming practices, the complex landscape structure and remote areas. The combined length of the road network is 429,074 km (2021), and the average road density is 1.36 km per km^2^ but is not uniform across the country: depending on the province, it can range from 0.9 to 2.0 km per km^2^ (https://stat.gov.pl). Speed limits are 140, 120 and 90 km/h for motorways, expressways and other roads, respectively, and 50 km/h within built-up areas. In recent years, the road network has been expanding rapidly: the length of motorways, for example, increased from 552 to 1,753 km between 2004 and 2022 and is still increasing (https://www.gov.pl).

### Data collection

This work is based on volunteer-collected data entered into the Polish Roadkill Observation System (PROS), which is a platform for recording and exchanging information on wildlife-vehicle collisions in Poland. Unlike other Polish roadkill data resources (collected by road maintenance personnel or police), PROS is the only nationwide programme covering all terrestrial vertebrate species. The programme was launched in June 2015, but observations dating back to 2000 can also be entered (Supplementary Table S1). Data is collected opportunistically and uploaded to the PROS database via a web application http://zwierzetanadrodze.pl, where any individual user (citizen scientist) can enter observations of any terrestrial vertebrate species encountered on road throughout Poland. Each observation is pin-located on a map giving geo-references while further checkboxes allow for detailed characteristics of the event.

To minimize limitations associated with collecting data by citizen scientists^51^ and to improve the quality of the database, a number of measures have been applied. The fundamental unit of the database, and the basis for subsequent analyses, was a single record; therefore, quality checks focused on the validity of individual entries. Each entry is reviewed by the biologists and portal administrators before acceptance, so that any discrepancies detected in the data are discussed with the author, corrected or removed. The discrepancies most often concern the choice of environments inconsistent with the place indicated on the map and the wrong number or type of road. Duplicate records were removed; however, they were extremely rare (only a few cases). Photo upload is encouraged to help in species identification. With difficult species or decayed carcasses, there is an option to assign the casualty to a higher class or genus. A checkbox-based design of the observation sheet with enlarged contextual help reduces the likelihood of error.

From the full dataset, we extracted the number of observed WVCs per species, the season of the observation (Spring, Summer, Autumn or Winter) and the dominant environment in the surrounding landscape where the record was observed. Records concerning pets and observations of live animals were excluded. Each record was classified based on the percentages of the different land use types, considering the CORINE land-cover (CLC) vector data derived from 25-m resolution satellite data^52^. Land-use categories level 2 taken from CLC were grouped to obtain the 6 main land-use types used in this study (i.e. arable land areas, industrial areas, urban fabric, artificial non-agricultural vegetated areas, permanent crops, mines and dumps areas). To classify the dominant environment, we calculated the percentage of each land-use type through the “intersect operator” of ArcGIS 10.8.1 software^53^ in a circle of 100 m radius around each observation. Then, each record was classified as the dominant environment depending on the land use type with a cover of > 60%^54^. Areas where none of the land-use types had at least 60%, were classified as “mixed” environments, obtaining a total of seven different types of dominant environments. Additionally, for each species, the average body mass from the literature (Supplementary Table S2) was recorded, and their distribution range in Poland was calculated as a percentage. The ranges of each species were taken from the distribution atlases of mammals (www.iop.krakow.pl/Ssaki/), herpetofauna (www.iop.krakow.pl/plazygady), and birds (www.ebba2.info). Finally, for each species, a value of carcass persistence was assigned, related to its body mass, based on the data indicated in the literature^55^.

### Data analysis

We focused on cross-taxonomic comparisons, so most analyses were conducted at the class level and presented comparatively. We used the observed number of WVCs per species as a quantitative index. The potential associations, strength and direction between observed road mortality of wildlife species in Poland with the season, dominant environment, the species body mass, distribution range in Poland and expected carcass persistence, were examined using a Generalized Linear Mixed Model (GLMM)^56^. In the GLMM, observed WVCs per species was log-transformed and used as the response variable with a normal distribution. Body mass, distribution range in Poland, carcass persistence, animal group (i.e., amphibians, reptiles, birds, and mammals), season, as well as the interaction between animal group and season were used as predictors. The dominant environments were included as random factors to control for possible consistent differences among land use types in the surroundings (groups = 7). The ID of observers was added as a random factor to mitigate any possible effect related to the different effort of single data collectors^57^(groups = 34). All observers collected more than 100 observations. A test of the variance inflation factor (VIF) of the model was applied to check for potential multicollinearity among predictors, using the package ‘fmsb’ for R^58^, and all predictors were incorporated into the model procedure because with VIF < 2 (a correlogram among them is shown in Supplementary Fig. S1). Quantitative variables were scaled before running the mixed model, using the function ‘scale’ in R. All statistical tests were performed with R software v. 4.1.2^59^. For the visualization of the data about WVCs for each of the fourth vertebrate groups, we chose a raincloud plot, using the ‘smplot2’ Package for R^60^. Plots showing the temporal distribution of road mortality were generated using Plotrix, a visualization package for R programming language^61^.

Finally, a chi-square test of independence or Fisher’s exact test (when the number of species was small) was performed to compare the proportion of endangered species in Poland with those recorded in PROS, providing a comparative view of the impact of roads on species endangerment among vertebrate classes. In the case of birds, the comparison included only species that regularly breed in Poland, rather than the total number of species recorded in the country. The latter also includes rare or accidental species observed only in a few instances, which are therefore unlikely to be detected on roads. The figures for species richness were based on recent national checklists^62^, www./komisjafaunistyczna.pl), and the number of threatened species followed the Polish Red Lists^63,64^.

## Results

### Numbers and composition of roadkills

For this study 19,028 records submitted by 412 observers and spanning the years 2000–2022, were used. Most of the data (90.8%) were from 2016 onwards (Supplementary Table S1). Each record represented one or more individuals, so the full dataset consisted of 28,709 individuals killed on roads.

**Table 1:** Summary of road mortality recorded in PROS up to the end of 2022 in vertebrate classes in Poland.

Animal group	No. of records (%)	No. of casualties (%)	No. of records with > 1 casualty (% records)	No. of casualties per record	Range
Mammals	10,728 (56.4)	10,910 (38.0)	152 (1.4)	1.02	1–12
Birds	6114 (32.1)	6406 (22.3)	116 (1.9)	1.05	1–105
Amphibians	1411 (7.4)	10,061 (35.0)	539 (38.2)	7.13	1–500
Reptiles	775 (4.1)	1332 (4.6)	86 (11.1)	1.72	1–91
Total	19,028 (100)	28,709 (100)	893 (4.7)	1.51	1–500

**Fig. 1:**
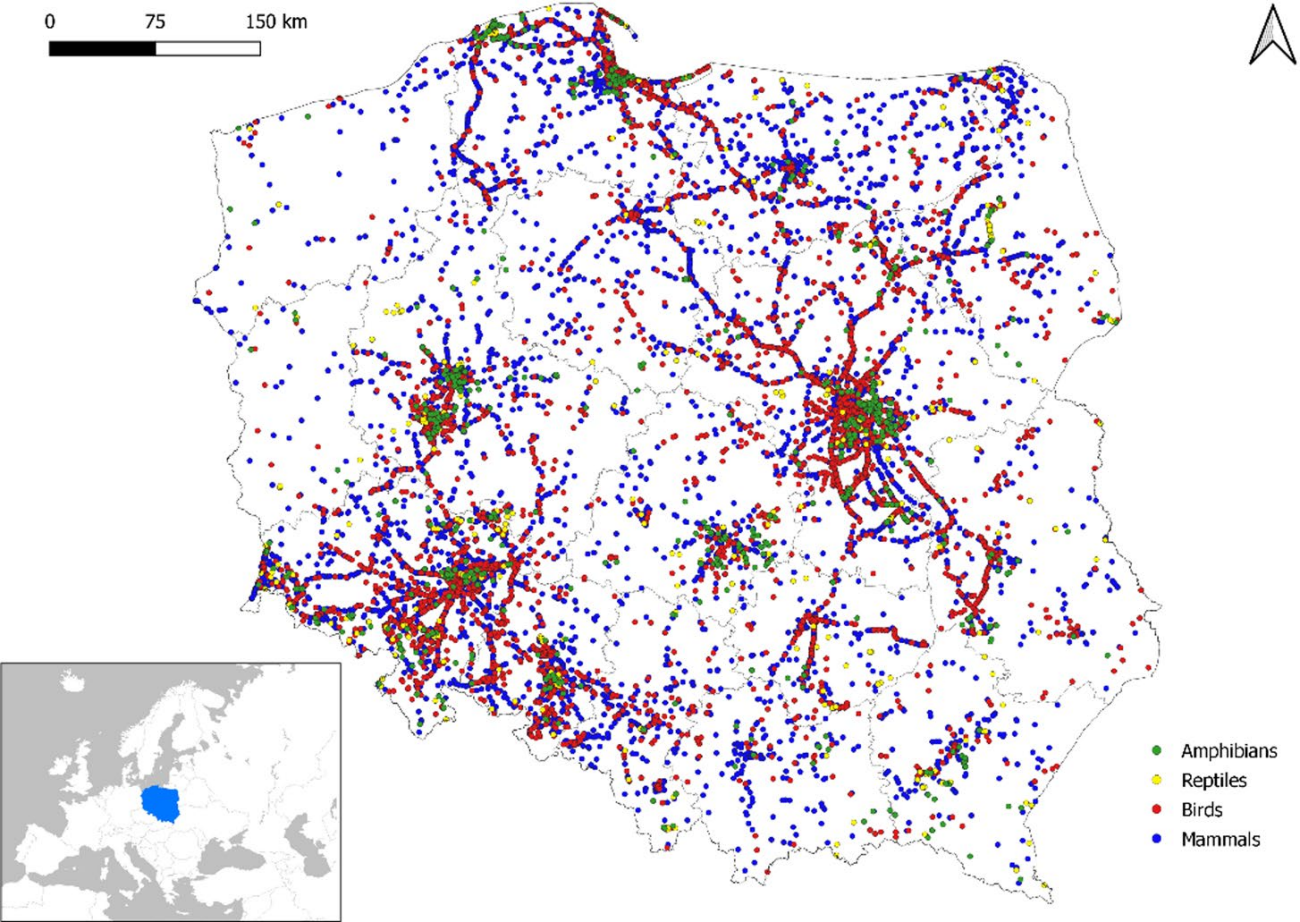
Distribution of wildlife-vehicle collisions entered into PROS until the end of 2022 (*N* = 19,004), divided into four vertebrate classes and superimposed on a map of the Polish provinces (voivodeships) (313,933 km²).

The observations were associated with the largest cities, while the west and east of the country were underrepresented, yet there was no apparent pattern of distribution in the vertebrate classes (Fig. 1). The classes differed in mortality magnitude: the approximate proportion of mammals to amphibians, birds and reptiles were 8:8:5:1; mammals and amphibians are thus the most frequently reported vertebrates by citizen scientists in Poland (Table 1).

**Table 2:** Number and percentages of total and threatened vertebrate species recorded in PROS and in Poland. Species richness in Poland according to Bogdanowicz (2014), Wilk et al. (2020), and www.komisjafaunistyczna.pl. Threatened species include the IUCN categories of CR, EN, VU, NT, RE, DD (Wilk et al. 2020, Głowaciński 2022; Supplementary Table S2). The significance of the proportions of threatened species in PROS and in Poland is given in the last two columns.

Animal group	No. species in PROS (threatened sp.)	No. species in Poland (threatened sp.)	% Polish species killed on roads	% threatened species killed on roads	% threatened species in Poland	chi square/Fisher’s exact test (df = 1)	*p*-value
Mammals	50 (7)	98 (31)	51.0	14.0	31.6	3.358	0.067
Birds (breeding)	125 (16)	230 (77)	54.3	13.0	33.5	11.079	0.001
Birds (total)	133	469	28.4				
Amphibians	14 (6)	18 (9)	77.8	42.9	50.0	0.059	0.808
Reptiles	8 (3)	12 (3)	66.7	37.5	25.0		1.000
Total – including breeding birds only	197 (32)	358 (120)	55.0	16.2	33.5	11.366	0.001
Total	205	597	34.2				

The 205 identified species recorded as traffic victims represent 34.2% of all the terrestrial vertebrate species occurring in Poland (*N* = 597, 2022) and 55% if we take only breeding birds into account (Table 2). Over 77% of amphibian species in Poland were recorded as roadkill. Approximately half of all mammals and breeding bird species in Poland were reported as roadkill, and 28.4% of the total species richness of birds, which also includes rarities, such as Red-footed Falcon (*Falco vespertinus*), Jacksnipe (*Lymnocryptes minimus*), Bearded Reedling (*Panurus biarmicus*), or Wood Duck (*Aix sponsa*) (Supplementary Table S2). Threatened species listed on the Polish Red Lists were recorded in all taxa, most frequently in herpetofauna. They include iconic species, such as European Bison (VU), Brown Bear (NT), Peregrine Falcon (*Falco peregrinus*) (VU), Fire Salamander (*Salamandra salamandra*) (NT), Aesculapian Snake (*Zamenis longissimus*) (CR), European Pond Turtle (*Emys orbicularis*) (EN) (Supplementary Table S2). The percentage of threatened species represented in WVCs as a percentage of these species in Poland did not differ among the herpetofauna, but was significantly lower in breeding birds and marginally significant in mammals (Table 2).

The 10 most abundant taxa accounted for 67.5% of the observed number of casualties and included seven species of mammals (including two hedgehog species taken together in the list), two species of amphibians, one bird and one reptile (Table 3). The dominance pattern was particularly pronounced among herpetofauna, where a single species dominated each group: the Common Toad (*Bufo bufo*) accounted for approximately 80% of all amphibian records, and the Grass Snake (*Natrix natrix*) for a similarly large share of reptile records.

**Fig. 2:**
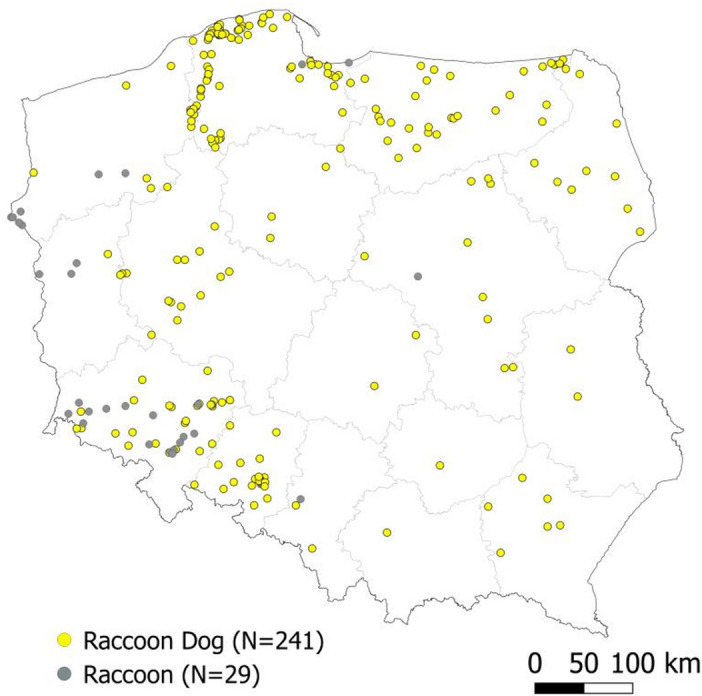
Records of Raccoon dog (*Nyctereutes procyonoides*) and Raccoon (*Procyon lotor*) collected in PROS.

Spatial patterns in the records reflect occurrence of species at a national scale, including the parapatric distribution of the two hedgehog species, the sympatric occurrence of the two marten species (www.iop.krakow.pl/Ssaki), differing expansion dynamics of invasive mammals (Fig. 2), and uneven population distributions of many other species (see maps in Supplementary Fig. S2).

**Table 3:** The ten most frequent traffic victims in Poland. Unidentified amphibians and unidentified birds have been omitted from the list. In Hedgehogs, both identified and unidentified carcasses are accounted for. The percentages were calculated after excluding the number of unidentified species in each class.

No.	Scientific name	Vernacular name	No of casualties	% casualties in taxonomic group
1	*Bufo bufo*	Common Toad	6705	79.11
2	*Erinaceus sp.*	Hedgehog	4013	37.50
3	*Vulpes vulpes*	Red Fox	1805	16.87
4	*Columba livia f. urbana*	Feral Pigeon	1114	18.83
5	*Natrix natrix*	Grass Snake	994	78.27
6	*Sciurus vulgaris*	Red Squirrel	718	6.71
7	*Rana temporaria*	Common Frog	716	8.45
8	*Capreolus capreolus*	Roe Deer	676	6.32
9	*Martes foina*	Beech Marten	531	4.96
10	*Meles meles*	Badger	529	4.94
	Total		17,801	67.53

### Multiple mortality

Although 95.3% of observations related to individual animals, in each taxonomic group, several casualties per record were also reported, including mass incidents. Mean numbers of individuals per record differed among taxa and were prominent in amphibians (Table 1). Indeed, 19 out of 20 submissions reporting at least 100 kills related to amphibians, mainly Common Toad. Mass incidents were also relatively common in reptiles, including 16 entries with more than 10 fatalities (max. 91), which related solely to Grass Snakes (Supplementary Table S3). Multiple fatalities in birds and mammals were incidental, but the extreme and recurrent road mortality of birds (up to 105 individuals of Robin (*Erithacus rubecula*)) on a road crossing the migration flyway along the Baltic coast cannot be ignored.

### Factors of road mortality

**Fig. 3:**
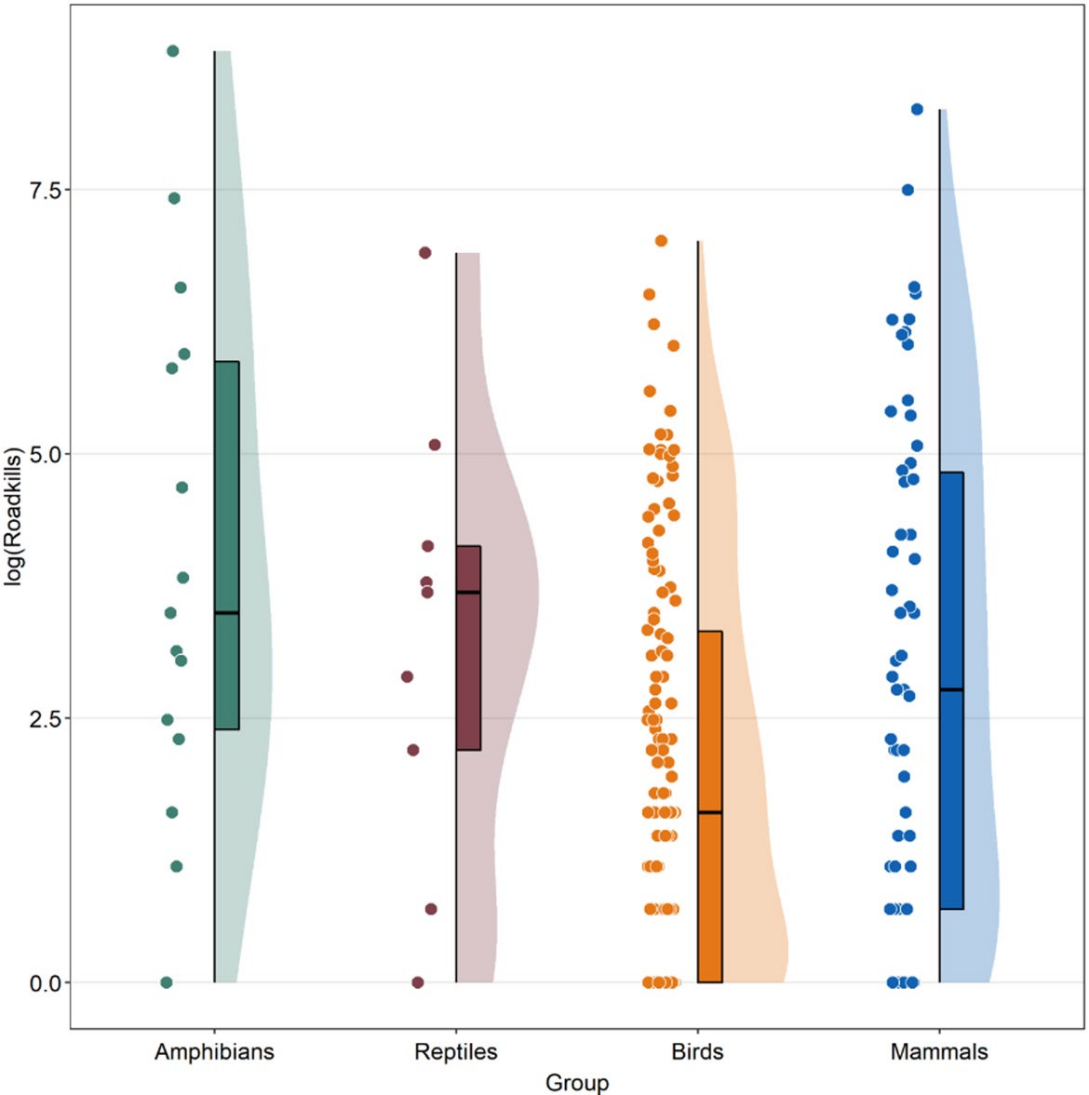
Number of observed wildlife-vehicle collisions (log scale on the y-axis) in the four vertebrate groups. The raincloud plot shows the raw data, probability density and summary statistics such as the median (black bar in the middle of the coloured rectangles), upper and lower quartiles by presenting individual data, a violin plot and a boxplot together.

**Fig. 4:**
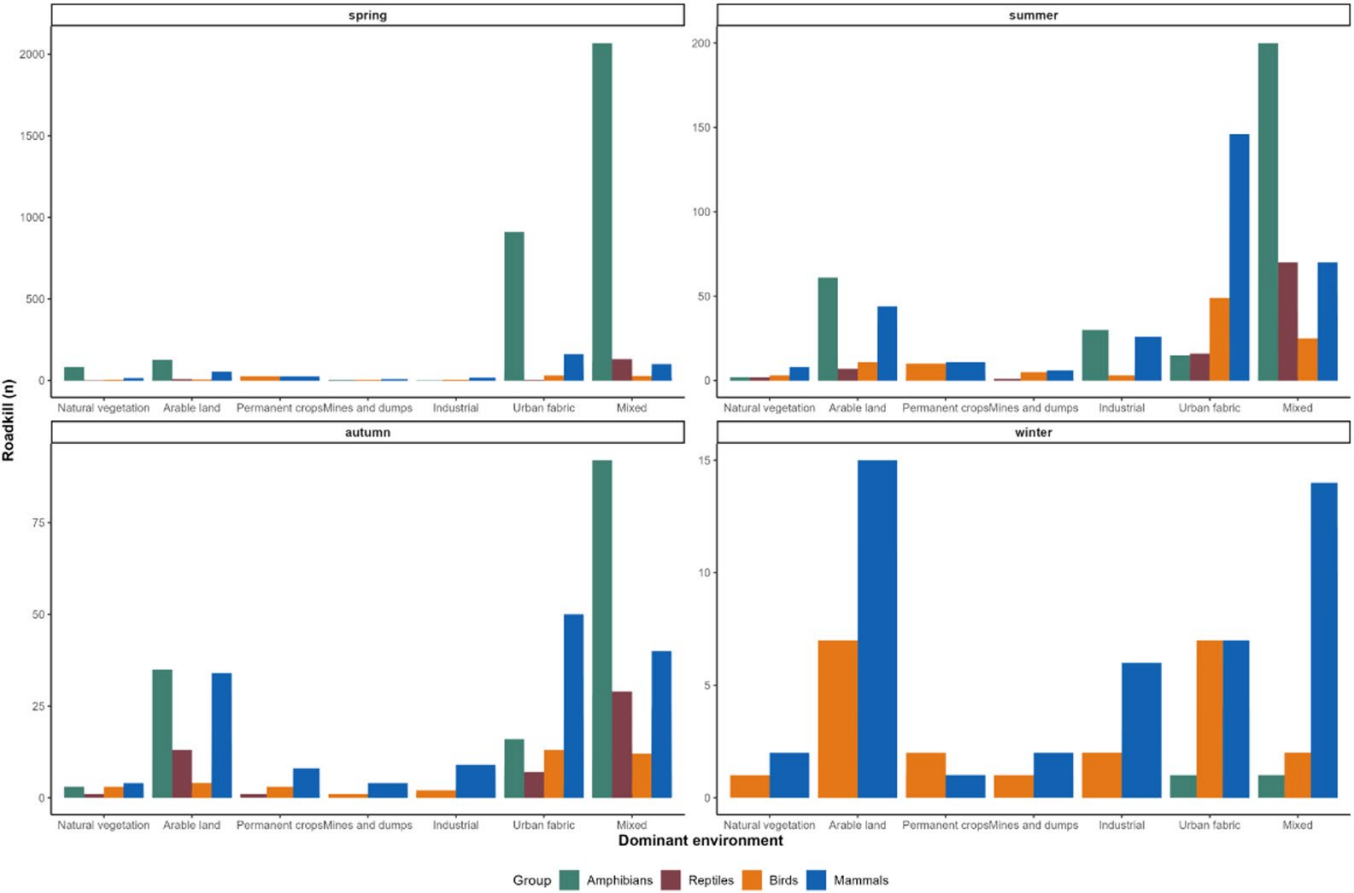
Number of observed wildlife-vehicle collisions in the four vertebrate groups, regarding the season and the type of dominant environment.

The number of observed WVCs per species was significantly lower for birds and mammals compared with amphibian or reptile species (Fig. 3). When considering the GLMM results, we found that observed roadkill was positively correlated with the body mass and carcass persistence of species (Table 4). The number of observations per species was relatively lower for birds, mammals and reptiles compared to amphibians (Table 4; Fig. 4), while such values for all groups increased significantly during the Spring, decreasing during the Winter (Table 4; Fig. 4). Finally, we found a relative reduction in observed WVCs for birds, mammals and reptiles during the Spring, with a positive flexion during Summer and Winter only for birds and mammals if compared with the other groups (Table 4; Fig. 4). The relationship between the observed WVCs and species distribution range in Poland was not significant. Considering main land-use types, arable lands, urban fabric and mixed environments occurred most often in the record surroundings, irrespective of season and class of vertebrates (Fig. 4).

**Fig. 5:**
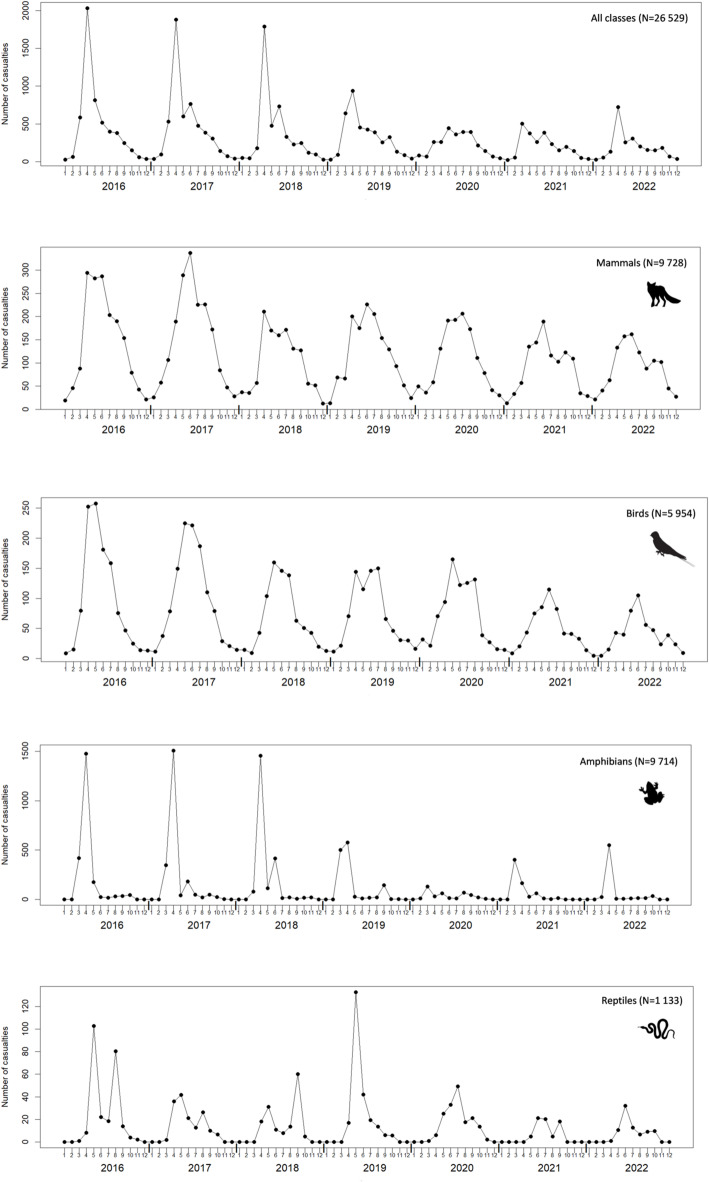
Temporal distribution of wildlife-vehicle collisions on roads in Poland, based on data entered into PROS in 2016–2022. Dots represent monthly totals of the observed number of casualties. All collisions, as well as collisions divided into four taxonomic groups, are presented on separate plots.

Spring-summer peaks and winter troughs produced a prominent sine wave distribution in WVCs consistent across years (Fig. 5). While this pattern applied to all taxonomic groups, it was most regular in mammals, birds and all classes combined. In amphibians, there was a very distinct, short-term peak in early spring and usually a second, much smaller one in late summer/autumn (Sept-Oct). The WVCs distributions of reptiles were less clear and also bi-modal in most years.

**Table 4:** Results of a generalized linear mixed model assessing the number of observed records per species in Poland spanning the years 2000–2022, concerning the species range of distribution in the country, the body mass, the carcass persistence and the interaction between animal group (i.e., amphibian, reptile, bird and mammal) and season. The dominant environment (groups = 7) and observer ID (groups = 34) were included as random factors to control for possible consistent differences among land use types in the surroundings and data collection efforts, respectively. Only significant variables are shown. Est = estimate, S.E. = standard error, t value = values of the statistic, d.f. = degree of freedom and p-value. Model performance: Pseudo-R^2^ = 0.23.

	Est.	S.E.	t value	d.f.	*p*-value
(Intercept)	0.584	0.147	4.0	41.4	0.000
Scaled body mass	−0.070	0.013	−5.6	4496.9	0.000
Scaled carcass persistence	0.084	0.015	5.6	4508.9	0.000
Group (Birds)	−0.739	0.119	−6.2	3566.6	0.000
Group (Mammals)	−0.338	0.113	−3.0	15320.8	0.003
Group (Reptiles)	−0.440	0.154	−2.9	51514.3	0.004
Season (Spring)	0.653	0.125	5.2	5399.7	0.000
Season (Winter)	−1.056	0.375	−2.8	4826.6	0.005
Group (Birds) : Season (Spring)	−0.437	0.138	−3.2	1640.6	0.002
Group (Mammals) : Season (Spring)	−0.519	0.133	−3.9	5467.7	0.000
Group (Reptiles) : Season (Spring)	−0.526	0.196	−2.7	25565.8	0.007
Group (Birds) : Season (Summer)	0.344	0.153	2.2	1660.9	0.025
Group (Mammals) : Season (Summer)	0.298	0.148	2.0	5897.7	0.045
Group (Birds) : Season (Winter)	0.976	0.385	2.5	4912.9	0.011
Group (Mammals) : Season (Winter)	0.800	0.380	2.1	4824.6	0.035

## Discussion

This first synthesis of wildlife–vehicle collisions (WVCs) in Poland highlights the considerable potential of citizen science for detecting broad-scale patterns that are rarely accessible through conventional roadkill surveys. The PROS database is unique among roadkill data sources in Poland due to its nationwide scope, taxonomic breadth, and extensive volunteer participation. As a result, the database captures a wider spectrum of traffic victims, including small species typically underrepresented in official statistics based on police or road service records. By contrast, systematic monitoring programmes, although methodologically rigorous, are generally limited in spatial scope and rarely offer cross-taxonomic comparisons. Of approximately 60 Polish publications addressing WVCs (listed on the PROS website), only six adopted a multi-taxon perspective.

The broad taxonomic converage of PROS enabled the detection of species not previously reported as traffic victims in Poland, including several rare and conservation-relevant taxa such as the Aesculapian snake, European bee-eater (*Merops apiaster*), Roller (*Coracias garrulus*), Shelduck (*Tadorna tadorna*)^65^. The database also provided noteworthy faunistic records, including one of the first documented occurrences of the golden jackal (*Canis aureus*) in Poland^66^ and records of the otherwise rare Red-footed Falcon during its invasion in 2015^67^.

Beyond quantifying mortality, PROS also proved valuable for documenting species distributions and dispersal routes. In particular, the data reflect ongoing biological invasions in Poland. Raccoon dog (*Nyctereutes procyonoides*), an earlier invader from the east, is now distributed throughout the country but remains most abundant in northern Poland^68^. By contrast, Raccoon (*Procyon lotor*) expanded from western Europe, with a marked increase in records only since the 1990s, and is currently concentrated in western Poland^69^. Finally, PROS revealed unexpectedly high levels of bird mortality along the Baltic coast, a major migratory corridor^70^. In particular, the European robin, a common forest-floor specialist, emerged as a frequent traffic victim, consistent with previous findings that small woodland birds are particularly vulnerable to traffic deaths^71^.

### Potential biases and data limitations

Despite its effectiveness in rapidly generating large datasets, the PROS initiative is subject to biases typical of volunteer-based monitoring. These include unequal detectability among taxa, overrepresentation of large or charismatic species, risks of misidentification, and spatial bias toward densely populated areas and roads with lower traffic speed^12,72,73^. To reduce these biases, quality-control measures were applied during data submission and analysis (see Methods). In particular, PROS follows a standardized validation protocol in which each record is reviewed by biologists prior to acceptance, allowing inaccurate or unreliable entries to be corrected or removed. At the analytical stage, accounting for variation associated with landscape context and data collectors helped ensure that the reported differences among vertebrate classes reflect general mortality patterns rather than artefacts of site-specific conditions or observer-related effects. Importantly, this study did not aim to estimate total national mortality or to identify mortality hotspots, analyses addressed elsewhere^74^, but rather to compare relative patterns across vertebrate classes. Within this framework, the results are consistent with other large-scale citizen science studies^25,75^ and are informative, provided that caution is exercised when extrapolating to absolute mortality levels.

### Taxonomic composition of roadkill

Marked differences were observed among vertebrate classes, with mammals and amphibians accounting for the majority of reported WVCs. The high representation of amphibians was expected given the widespread availability of suitable habitats in Poland and the findings of previous local studies^46,76–80^, particularly in wetland-rich areas where amphibians may comprise over 90% of recorded casualties^40,81^. Mass mortality events during seasonal migrations strongly inflated amphibian counts and produced pronounced temporal clustering, thus blurring the overall picture of vertebrate mortality. Common toad and true frogs (*Rana* ssp.) were consistently the most frequently recorded species in Polish road studies.

Mammal data are less affected by such episodic events, although detectability biases remain important. Large-bodied mammals are overrepresented, whereas small species are frequently overlooked^73^. Nevertheless, rodents and other small mammals are known to experience high road mortality^24,42,82^, especially in agricultural landscapes that dominate much of Poland. These patterns suggest that mammal mortality on roads is substantial, even if not fully captured by citizen science data.

Birds were recorded less frequently than mammals and amphibians, a pattern consistent with previous Polish studies^40,81,^ but likely reflecting underdetection rather than true mortality levels. Bird collisions are expected to be common in Poland’s predominantly lowland and open landscapes^83^, and carcass persistence and detectability is comparable to that of amphibians for species of similar size^22^. However, many birds hit by cars are more difficult or even impossible to detect, as they get thrown beyond the line of the road or become trapped on car fronts^31,73^. Moreover, birds are active year-round, further supporting the inference that their contribution to total road mortality is high.

Reptiles constituted the smallest proportion of records. This result reflects their limited species richness in Poland (12 species), localized distributions^84^, rapid carcass removal^22^, and prolonged periods of inactivity. Nevertheless, low representation in multi-taxon datasets should not be interpreted as low vulnerability. Evidence from Poland and other regions indicates that reptiles, particularly snakes, can experience severe localized road mortality with potentially strong demographic consequences^23,25,28^.

### Multiple mortality events

Although most reports of multiple mortality involved amphibians, such events were documented in all vertebrate classes. Particularly striking were mass mortalities of Grass Snakes along roads intersecting well-populated habitats, sometimes involving hundreds of individuals within short periods. The frequent involvement of gravid females suggests disproportionate impacts on population reproductive output^85,86^, especially if such events recur annually (for examples see Supplementary Table S3). Notably, all documented reptile mass mortality sites were located near habitats of high conservation value, underscoring the significance of WVCs even in protected areas.

In contrast, multiple mortalities of birds and mammals were typically associated with specific circumstances (such as family groups or flocks foraging on roads and being startled by traffic) rather than with habitat quality per se^87^. PROS records further suggest higher mortality on winding roads, at higher speeds, and in collisions involving heavy vehicles, although additional targeted data are required to confirm these patterns. For example, studies conducted in Spain did not reveal a significant relationship between bird and mammal collisions and road tortuosity or fencing, but rather with distance to water sources and air temperature^10^.

### Spatial and temporal patterns

Modeling results revealed a strong positive relationship between animal body mass and recorded roadkill frequency, consistent with the well-established influence of carcass detectability^88^. Surprisingly, no significant association was observed between WVCs frequency and species’ distribution range within Poland, suggesting that road mortality is more strongly driven by other factors, such as local road characteristics, landscape context, or species-specific behavioral traits than by a species’ range size. Indeed, several examples indicate that vulnerability to road collisions may vary independently of abundance. For instance, the skylark (*Alauda arvensis*), Poland’s most abundant bird species, was rarely recorded (14 carcasses), whereas comparatively rare species such as the Raccoon dog and the Elk (*Alces alces*) appeared with unexpectedly high frequencies (222 and 55 records, respectively; Supplementary Table S2).

WVCs frequency showed strong seasonal and weak interannual variation. Low winter mortality and sharp increases during warmer months mirror animal activity patterns linked to reproduction, dispersal, and migration^89,90^. Mammal mortality peaked in spring and early summer, coinciding with mating and juvenile dispersal, while bird mortality formed a broad peak spanning spring migration, breeding, and fledging periods. Contrary to expectations, autumn migration was not associated with increased bird mortality, possibly due to its more diffuse spatial and temporal nature^91^.

Amphibians exhibited pronounced mortality peaks during spring migrations to breeding sites, indicating severe nationwide losses each year, although population-level effects remain difficult to quantify. Importantly, the first amphibian records each year may serve as useful indicators of phenological shifts. In 2020, the first Common Toad and Moor Frog (*Rana arvalis*) killed on roads were reported as early as 4 February, reflecting the extremely mild winter of 2019/2020. Reptile mortality fluctuated across spring and summer, reflecting mating movements, egg-laying migrations, and the dispersal of hatchlings^85,92^.

### Tracking road mortality with citizen observations

This study highlights the value of citizen science for understanding wildlife–human interactions and for advancing road ecology and conservation research. Despite its unstandardized nature, data contributed to PROS provide an efficient means of identifying broad-scale mortality patterns. A key advantage is the rapid collection of spatially extensive data across diverse regions and habitats at minimal cost. In countries with dense road networks, such as Poland, citizen-collected data on traffic mortality therefore represent a particularly important resource for conservation science.

From a conservation perspective, 55% of terrestrial vertebrate species recorded in Poland were documented as road victims. Given the short duration of the PROS project and the opportunistic nature of data collection, this proportion is remarkably high and includes rare and charismatic species not previously reported as roadkill. Notably, 16% of the recorded species are classified as threatened, including most red-listed amphibians and reptiles. Although absolute mortality numbers may appear low, many of these species have small effective population sizes, making them vulnerable to additional road-related mortality. Importantly, numerous collisions involving threatened species, including several mass mortality events, occurred within protected areas, indicating that existing conservation measures may be insufficient (Supplementary Table S3).

### Conclusions

Using a nationwide citizen-science dataset, this study provides the first comprehensive overview of traffic collisions with wild terrestrial vertebrates in Poland and establishes a baseline for future research and mitigation planning. Our results demonstrate that road mortality is widespread across the country and affects a broad taxonomic spectrum, including rare and threatened species. The cross-taxonomic and multi-year approach further reveals marked differences in WVCs among vertebrate classes, driven largely by body mass and detectability of species, and seasonal activity patterns. Although many of these processes have been discussed in road ecology literature, few studies have documented them simultaneously at broad taxonomic and geographical scales.

To advance toward more accurate and policy-relevant nationwide assessments, future research should integrate multiple approaches to studying WVCs. In particular, this involves moving from opportunistic reporting toward a nationally coordinated, systematic survey based on predefined road segments that are spatially representative of the country (e.g. land covers, road classes, traffic levels, and biogeographical regions). Segments would be randomly selected within these strata and assigned to trained volunteers who survey the same segment at a fixed frequency, ideally weekly, using standardized methods. Volunteers would follow the same procedures as the PROS (GPS location, species or taxonomic group, carcass condition, date and time, weather conditions, and take a photo for validation, while noting survey effort (distance, duration, method) to allow calculation of mortality per kilometre). We recommend a pilot in a few regions would test the approach before scaling nationally, producing robust, spatially representative data for hotspot identification.

## Electronic Supplementary Material

Below is the link to the electronic supplementary material.

Supplementary Material 1

## Data Availability

The datasets used and/or analysed during the current study available from the corresponding author on reasonable request.
