# Supplementary material for: Patterns of wildlife-vehicle collisions in Poland: a cross-taxonomic analysis of a nationwide citizen science dataset
Source: Sci Rep. 2026 May 12;16:20754. doi: 10.1038/s41598-026-52546-z (PMC13338236; doi:10.1038/s41598-026-52546-z)
Supplement: Supplementary file 1 — Supplementary Material 1 [file 41598_2026_52546_MOESM1_ESM.pdf]

Patterns of wildlife-vehicle collisions in Poland: a cross-taxonomic analysis of a nationwide citizen science dataset

Andrzej Wuczyński, Karol Kustusch, Przemysław Stolarz, Maciej Wuczyński, Clara Grilo, Tomé Neves, Federico Morelli

Electronic Supplementary Material

Table S1

Summary of data entered into the PROS up to the end of 2022. Figures represent the number of casualties and the number of records by vertebrate class and year.

Variable	Animal group	pre-2016	2016	2017	2018	2019	2020	2021	2022	2016-2022	Total
**No of casualties**	Mammals	1182	1734	1818	1238	1427	1323	1104	1083	9728	10910
	Amphibians	347	2242	2236	2149	1325	407	698	657	9714	10061
	Birds	452	1147	1182	819	862	876	573	495	5954	6406
	Reptiles	199	259	159	148	242	172	70	83	1133	1332
	All classes	2180	5382	5395	4354	3856	2778	2445	2318	26529	28709
**No of records**	Mammals	1167	1699	1787	1219	1396	1306	1088	1066	9561	10728
	Amphibians	72	287	297	209	166	202	101	77	1339	1411
	Birds	440	979	1131	808	845	861	560	490	5674	6114
	Reptiles	76	120	107	111	103	134	64	60	699	775
	All classes	1755	3085	3322	2347	2510	2503	1813	1693	17273	19028

Table S2

Species composition of animal roadkill in Poland by vertebrate classes, based on data entered into the PROS until the end of 2022. Red List categories follow the Polish Red Lists of threatened animals (Wilk et al. 2020, Głowaciński 2022). Body masses are the average of the min-max values obtained from Pucek (1984), Juszczyk (1987), Najbar (2006), Storchová & Hořák (2018), and Najbar et al. (2022). * - bird species that do not breed in Poland

No.	Latin name	Vernacular name	Red List category	Body mass	No. of records	% of records	No. of roadkills	% of roadkills
	**Mammals**							
1	*Erinaceus sp.*	Hedgehog		806.5	3765	35.1	3856	35.3
2	*Vulpes vulpes*	Red Fox		6000.0	1788	16.7	1805	16.5
3	*Sciurus vulgaris*	Red Squirrel		307.5	716	6.7	718	6.6
4	*Capreolus capreolus*	Roe Deer		16500.0	675	6.3	676	6.2
5	*Martes foina*	Beech Marten		1694.5	529	4.9	531	4.9
6	*Meles meles*	Badger		10850.0	523	4.9	529	4.8
7	*Lepus europaeus*	Hare		4250.0	466	4.3	469	4.3
8	*Martes sp.*	Marten		1664.5	456	4.3	458	4.2
9	*Sus scrofa*	Wild Boar		130500.0	214	2.0	247	2.3
10	*Nyctereutes procyonoides*	Racoon Dog		7200.0	221	2.1	222	2.0
11	*Martes martes*	Pine Marten		1140.0	159	1.5	160	1.5
12	*Erinaceus roumanicus*	White-breasted Hedgehog		735.0	130	1.2	136	1.2
13	*Mustela putorius*	Polecat		1180.0	126	1.2	127	1.2
14	*Talpa europaea*	Mole		95.0	111	1.0	117	1.1
15	*Rattus norvegicus*	Brown Rat		315.5	113	1.1	114	1.0
16	*Mustela nivalis*	Least Weasel		82.0	66	0.6	69	0.6
17	*Lutra lutra*	Otter		6300.0	69	0.6	69	0.6
18	*Alces alces*	Elk	NT	288500.0	55	0.5	55	0.5
19	*Castor fiber*	Beaver		26500.0	41	0.4	41	0.4
20	*Cervus elaphus*	Red Deer		178000.0	34	0.3	35	0.3
21	*Apodemus agrarius*	Striped Field Mouse		23.5	31	0.3	33	0.3
22	*Cricetus cricetus*	Hamster	VU	555.0	33	0.3	33	0.3
23	*Procyon lotor*	Racoon		8000.0	20	0.2	22	0.2
24	*Erinaceus europaeus*	European Hedgehog		816.0	21	0.2	21	0.2
25	*Mustela erminea*	Stoat		171.0	18	0.2	18	0.2
26	*Rodentia*	Rodents		103.0	16	0.1	16	0.1
27	*Mustela vison*	American Mink		990.0	16	0.1	16	0.1
28	*Sorex araneus*	Common Shrew		10.5	15	0.1	15	0.1
29	*Ondatra zibethicus*	Muskrat		1100.0	10	0.1	10	0.1

30	*Canis lupus*	Wolf	NT	51000.0	9	0.1	9	0.1
31	*Chiroptera*	Bats		23.6	9	0.1	9	0.1
32	*Apodemus flavicollis*	Yellow-necked Field Mouse		30.0	9	0.1	9	0.1
33	*Soricomorpha*	Soricomorpha		9.3	6	0.1	7	0.1
34	*Glis glis*	Edible Dormouse	NT	61.5	5	0.0	5	0.0
35	*Neomys fodiens*	Water Shrew		17.4	4	0.0	4	0.0
36	*Bison bonasus*	European Bison	VU	620000.0	4	0.0	4	0.0
37	*Sorex minutus*	Pigmy Shrew		4.2	3	0.0	3	0.0
38	*Apodemus sylvaticus*	Wood Mouse		20.0	3	0.0	3	0.0
39	*Eptesicus serotinus*	Serotine Bat		24.0	3	0.0	3	0.0
40	*Pipistrellus sp.*	Pipistrelle		6.4	3	0.0	3	0.0
41	*Arvicola amphibius*	Water Vole		130.0	2	0.0	2	0.0
42	*Muscardinus avellanarius*	Hazel Dormouse		16.0	2	0.0	2	0.0
43	*Nyctalus noctula*	Common Noctule		30.5	2	0.0	2	0.0
44	*Myodes glareolus*	Bank Vole		24.3	2	0.0	2	0.0
45	*Dama dama*	Fallow Deer		59500.0	2	0.0	2	0.0
46	*Oryctolagus cuniculus*	European Rabbit		1750.0	2	0.0	2	0.0
47	*Microtus arvalis*	Common Vole		27.5	2	0.0	2	0.0
48	*Ursus arctos*	Brown Bear	NT	262000.0	1	0.0	1	0.0
49	*Plecotus auritus*	Brown Long-eared Bat		8.0	1	0.0	1	0.0
50	*Lynx lynx*	Lynx	NT	25000.0	1	0.0	1	0.0
51	*Micromys minutus*	Harvest Mouse		7.5	1	0.0	1	0.0
52	*Canis aureus*	Golden Jackal		10500.0	1	0.0	1	0.0
53	*Myotis daubentonii*	Daubenton’s Bat		7.3	1	0.0	1	0.0
54	*Mus musculus*	House Mouse		15.5	1	0.0	1	0.0
55	*Pipistrellus pipistrellus*	Common Pipistrelle		6.4	1	0.0	1	0.0
56	*Myotis mystacinus*	Whiskered Bat		4.0	1	0.0	1	0.0
	Unidentified mammals			555.0	210	2.0	210	1.9
	Total mammals				10728	100.0	10910	100.0
	**Birds**							
1	*Columba livia f. urbana*	Feral Pigeon		334.0	1101	18.0	1114	17.4
2	*Turdus merula*	Blackbird		97.0	501	8.2	505	7.9
3	*Passer domesticus*	House Sparrow		29.3	381	6.2	412	6.4
4	*Erithacus rubecula*	Robin		17.6	149	2.4	269	4.2
5	*Columba palumbus*	Wood Pigeon		496.5	221	3.6	223	3.5
6	*Corvus monedula*	Jackdaw		236.0	178	2.9	179	2.8
7	*Buteo buteo*	Common Buzzard		806.5	176	2.9	178	2.8
8	*Passer sp.*	Sparrow		26.0	162	2.6	169	2.6
9	*Turdus philomelos*	Song Thrush		76.0	154	2.5	155	2.4
10	*Streptopelia decaocto*	Collared Dove		195.0	154	2.5	154	2.4
11	*Turdus pilaris*	Fieldfare		105.1	148	2.4	154	2.4
12	*Hirundo rustica*	Barn Swallow		19.0	144	2.4	154	2.4
13	*Phasianus colchicus*	Pheasant		1134.0	147	2.4	148	2.3
14	*Sturnus vulgaris*	Starling		80.5	142	2.3	146	2.3
15	*Strix aluco*	Tawny Owl		495.5	132	2.2	132	2.1
16	*Passer montanus*	Tree Sparrow		22.8	112	1.8	121	1.9

17	*Garrulus glandarius*	Jay		164.4	116	1.9	118	1.8
18	*Anas platyrhynchos*	Mallard		1119.0	107	1.8	115	1.8
19	*Emberiza citrinella*	Yellowhammer		26.8	93	1.5	93	1.5
20	*Corvus frugilegus*	Rook	VU	459.0	71	1.2	88	1.4
21	*Corvus cornix*	Hooded Crow		526.8	80	1.3	83	1.3
22	*Fringilla coelebs*	Common Chaffinch		22.2	80	1.3	82	1.3
23	*Parus major*	Great Tit		18.3	70	1.1	72	1.1
24	*Dendrocopos major*	Great-spotted Woodpecker		74.0	63	1.0	64	1.0
25	*Pica pica*	Magpie		228.0	57	0.9	58	0.9
26	*Lanius collurio*	Red-backed Shrike		31.2	54	0.9	54	0.8
27	*Chroicocephalus ridibundus*	Black-headed Gull		267.5	50	0.8	50	0.8
28	*Asio otus*	Long-eared Owl		255.5	49	0.8	49	0.8
29	*Larus argentatus*	Herring Gull		1054.0	42	0.7	42	0.7
30	*Motacilla alba*	White Wagtail		20.5	39	0.6	40	0.6
31	*Perdix perdix*	Grey Partridge		382.0	35	0.6	37	0.6
32	*Carduelis cannabina*	Linnet		19.3	31	0.5	33	0.5
33	*Sylvia atricapilla*	Blackcap		18.6	31	0.5	31	0.5
34	*Carduelis carduelis*	Goldfinch		15.5	27	0.4	28	0.4
35	*Cyanistes caeruleus*	Blue Tit		11.5	26	0.4	27	0.4
36	*Chloris chloris*	Greenfinch		26.3	25	0.4	26	0.4
37	*Picus viridis*	Green Woodpecker		192.5	26	0.4	26	0.4
38	*Delichon urbicum*	House Martin		19.5	17	0.3	23	0.4
39	*Phoenicurus ochruros*	Black Redstart		16.0	21	0.3	22	0.3
40	*Ciconia ciconia*	White Stork		3245.5	22	0.4	22	0.3
41	*Sylvia communis*	Whitethroat		14.8	18	0.3	18	0.3
42	*Fringilla montifringilla*	Brambling*		22.9	2	0.0	18	0.3
43	*Sylvia curruca*	Lesser Whitethroat		12.4	16	0.3	16	0.2
44	*Alauda arvensis*	Skylark		38.5	14	0.2	14	0.2
45	*Larus canus*	Common Gull	VU	394.5	14	0.2	14	0.2
46	*Accipiter nisus*	Sparrowhawk		204.0	13	0.2	13	0.2
47	*Riparia riparia*	Sand Martin		13.5	6	0.1	12	0.2
48	*Coccothraustes coccothraustes*	Hawfinch		57.9	12	0.2	12	0.2
49	*Scolopax rusticola*	Woodcock		312.0	12	0.2	12	0.2
50	*Apus apus*	Swift		40.0	12	0.2	12	0.2
51	*Caprimulgus europaeus*	Nightjar		71.5	11	0.2	11	0.2
52	*Larus sp.*	Gull		839.7	11	0.2	11	0.2
53	*Emberiza calandra*	Corn Bunting		47.2	9	0.1	10	0.2
54	*Cygnus olor*	Mute Swan		11150.0	10	0.2	10	0.2
55	*Falco tinnunculus*	Kestrel		234.0	10	0.2	10	0.2
56	*Phoenicurus phoenicurus*	Redstart		15.7	9	0.1	9	0.1
57	*Troglodytes troglodytes*	Wren		8.9	7	0.1	9	0.1
58	*Turdus viscivorus*	Mistle Thrush		117.7	9	0.1	9	0.1
59	*Sitta europaea*	Nuthatch		23.1	8	0.1	8	0.1
60	*Fulica atra*	Coot		732.5	8	0.1	8	0.1
61	*Motacilla flava*	Yellow Wagtail		16.5	8	0.1	8	0.1

62	*Spinus spinus*	Siskin		13.8	7	0.1	7	0.1
63	*Saxicola rubetra*	Whinchat	NT	16.6	6	0.1	6	0.1
64	*Jynx torquilla*	Wryneck		40.0	6	0.1	6	0.1
65	*Alcedo atthis*	Kingfisher		43.5	6	0.1	6	0.1
66	*Acrocephalus palustris*	Marsh Warbler		12.0	6	0.1	6	0.1
67	*Poecile palustris*	Marsh Tit		10.5	6	0.1	6	0.1
68	*Aegithalos caudatus*	Long-tailed Tit		8.5	5	0.1	5	0.1
69	*Botaurus stellaris*	Bittern	NT	1231.0	5	0.1	5	0.1
70	*Muscicapa striata*	Spotted Flycatcher		16.3	5	0.1	5	0.1
71	*Phylloscopus trochilus*	Willow Warbler		9.1	5	0.1	5	0.1
72	*Vanellus vanellus*	Northern Lapwing	EN	218.5	5	0.1	5	0.1
73	*Ficedula hypoleuca*	Pied Flycatcher	NT	13.2	5	0.1	5	0.1
74	*Gallinula chloropus*	Moorhen		348.5	5	0.1	5	0.1
75	*Tyto alba*	Barn Owl	DD	354.0	5	0.1	5	0.1
76	*Serinus serinus*	Serin		12.0	4	0.1	4	0.1
77	*Corvus corax*	Raven		1217.5	4	0.1	4	0.1
78	*Lullula arborea*	Woodlark		26.5	4	0.1	4	0.1
79	*Acrocephalus schoenobaenus*	Sedge Warbler		11.9	4	0.1	4	0.1
80	*Regulus regulus*	Goldcrest		5.4	3	0.0	3	0.0
81	*Lanius excubitor*	Great Grey Shrike		67.0	3	0.0	3	0.0
82	*Ixobrychus minutus*	Little Bittern	DD	147.5	3	0.0	3	0.0
83	*Acrocephalus arundinaceus*	Great Reed Warbler		30.3	3	0.0	3	0.0
84	*Regulus ignicapilla*	Firecrest		5.2	3	0.0	3	0.0
85	*Dryocopus martius*	Black Woodpecker		291.0	3	0.0	3	0.0
86	*Athene noctua*	Little Owl	DD	153.0	2	0.0	2	0.0
87	*Dendrocopos minor*	Lesser Spotted Woodpecker		21.5	2	0.0	2	0.0
88	*Merops apiaster*	Bee-eater		55.0	2	0.0	2	0.0
89	*Tetrastes bonasia*	Hazel Grouse		397.5	2	0.0	2	0.0
90	*Luscinia megarhynchos*	Nightingale		20.2	2	0.0	2	0.0
91	*Coturnix coturnix*	Quail	VU	104.6	2	0.0	2	0.0
92	*Ardea cinerea*	Grey Heron		1433.0	2	0.0	2	0.0
93	*Pyrrhula pyrrhula*	Bullfinch		31.0	2	0.0	2	0.0
94	*Phylloscopus collybita*	Common Chiffchaff		7.7	2	0.0	2	0.0
95	*Rallus aquaticus*	Water Rail		129.5	2	0.0	2	0.0
96	*Anthus trivialis*	Tree Pipit		22.2	2	0.0	2	0.0
97	*Acrocephalus scirpaceus*	Reed Warbler		11.8	2	0.0	2	0.0
98	*Accipiter gentilis*	Northern Goshawk		931.5	2	0.0	2	0.0
99	*Falco vespertinus*	Red-footed Falcon*	RE	158.5	2	0.0	2	0.0
100	*Bombycilla garrulus*	Bohemian Waxwing*		62.4	1	0.0	1	0.0
101	*Anser fabalis*	Taiga Bean Goose*		2770.0	1	0.0	1	0.0
102	*Strix uralensis*	Ural Owl		765.0	1	0.0	1	0.0
103	*Picus canus*	Grey-headed Woodpecker		136.5	1	0.0	1	0.0
104	*Larus marinus*	Great Black-backed Gull*		1658.5	1	0.0	1	0.0
105	*Grus grus*	Crane		6055.0	1	0.0	1	0.0

106	*Aegolius funereus*	Tengmalm's Owl	NT	135.0	1	0.0	1	0.0
107	*Oenanthe oenanthe*	Northern Wheatear		23.0	1	0.0	1	0.0
108	*Cuculus canorus*	Common Cuckoo		111.0	1	0.0	1	0.0
109	*Oriolus oriolus*	Golden Oriole		69.8	1	0.0	1	0.0
110	*Tadorna tadorna*	Common Shelduck		1106.0	1	0.0	1	0.0
111	*Prunella modularis*	Dunnock		21.3	1	0.0	1	0.0
112	*Aix galericulata*	Mandarin Duck		570.0	1	0.0	1	0.0
113	*Emberiza hortulana*	Ortolan Bunting	VU	23.8	1	0.0	1	0.0
114	*Alopochen aegyptiaca*	Egyptian Goose		2270.0	1	0.0	1	0.0
115	*Upupa epops*	Eurasian Hoopoe		65.0	1	0.0	1	0.0
116	*Emberiza schoeniclus*	Common Reed Bunting		18.8	1	0.0	1	0.0
117	*Actitis hypoleucos*	Sandpiper		48.0	1	0.0	1	0.0
118	*Crex crex*	Corncrake	VU	163.0	1	0.0	1	0.0
119	*Columba oenas*	Stock Dove		292.0	1	0.0	1	0.0
120	*Falco peregrinus*	Peregrine Falcon	VU	893.0	1	0.0	1	0.0
121	*Galerida cristata*	Crested Lark		46.0	1	0.0	1	0.0
122	*Sylvia nisoria*	Barred Warbler		24.4	1	0.0	1	0.0
123	*Erythrina erythrina*	Common Rosefinch		23.4	1	0.0	1	0.0
124	*Lymnocryptes minimus*	Jacksnipe*	RE	55.0	1	0.0	1	0.0
125	*Saxicola rubicola*	European Stonechat		14.5	1	0.0	1	0.0
126	*Turdus iliacus*	Redwing	EN	62.9	1	0.0	1	0.0
127	*Mergus merganser*	Goosander		1435.5	1	0.0	1	0.0
128	*Aix sponsa*	Wood Duck*		725.0	1	0.0	1	0.0
129	*Luscinia luscinia*	Thrush Nightingale	NT	26.0	1	0.0	1	0.0
130	*Loxia curvirostra*	Red Crossbill		41.7	1	0.0	1	0.0
131	*Podiceps cristatus*	Great Crested Grebe		673.5	1	0.0	1	0.0
132	*Panurus biarmicus*	Bearded Reedling		14.7	1	0.0	1	0.0
133	*Dendrocopos medius*	Middle Spotted Woodpecker		58.0	1	0.0	1	0.0
134	*Buteo lagopus*	Rough-legged Buzzard*		947.5	1	0.0	1	0.0
135	*Larus fuscus*	Lesser Black-backed Gull		824.0	1	0.0	1	0.0
	Unidentified birds			62.8	486	7.9	491	7.7
	Total birds			271.6	6114	100.0	6406	100.0
	**Amphibians**							
1	*Bufo bufo*	Common Toad		85.8	906	64.2	6705	66.6
2	*Rana temporaria*	Common Frog		54.6	74	5.2	716	7.1
3	*Bufotes viridis*	Green Toad		53.2	71	5.0	381	3.8
4	*Pelobates fuscus*	Spadefoot	NT	29.0	31	2.2	334	3.3
5	*Pelophylax esculentus complex*	'Water Frogs'		129.0	56	4.0	108	1.1
6	*Rana sp.*	'Brown frogs'		53.0	31	2.2	74	0.7
7	*Rana arvalis*	Moor frog		15.8	26	1.8	46	0.5
8	*Lissotriton vulgaris*	Smooth Newt		2.1	20	1.4	33	0.3
9	*Pelophylax ridibundus*	Marsh Frog		141.0	3	0.2	23	0.2
10	*Triturus cristatus*	Great Crested Newt	NT	9.4	8	0.6	21	0.2
11	*Salamandra salamandra*	Fire Salamander	NT	26.1	8	0.6	12	0.1

12	*Bombina bombina*	Fire-bellied Toad	VU	8.0	2	0.1	10	0.1
13	*Pelophylax esculentus*	Edible frog		76.0	5	0.4	5	0.0
14	*Hyla sp.*	"Tree Frogs"	NT	6.6	4	0.3	4	0.0
15	*Epidalea calamita*	Natterjack Toad		22.5	3	0.2	3	0.0
16	*Lissotriton montandoni*	Carpathian Newt	NT	2.9	1	0.1	1	0.0
	Unidentified amphibians			27.6	162	11.5	1585	15.8
	Total amphibians			71.9	1411	100.0	10061	100.0
	**Reptiles**							
1	*Natrix natrix*	Grass Snake		185.5	514	66.3	994	74.6
2	*Anguis fragilis*	Slow Worm		44.2	145	18.7	162	12.2
3	*Lacerta agilis*	Sand Lizard		14.5	44	5.7	44	3.3
4	*Vipera berus*	Adder		106.0	40	5.2	40	3.0
5	*Zootoca vivipara*	Viviparous Lizard		6.0	15	1.9	18	1.4
6	*Coronella austriaca*	Smooth Snake	VU	80.4	8	1.0	9	0.7
7	*Emys orbicularis*	European Pond Turtle	EN	646.5	2	0.3	2	0.2
8	*Zamenis longissimus*	Aesculapian snake	CR	620.0	1	0.1	1	0.1
	Unidentified reptiles			93.2	6	0.8	62	4.7
	Total reptiles				775	100.0	1332	100.0
	**Total four classes**				19028		28709	

Table S3

List of five PROS entries with the highest number of fatalities in the vertebrate classes. Details of the collision, references reporting roadkills in the same locality and protection status of adjoining area are also listed, if available. The list proves that multiple collisions frequently occur in areas of conservation importance, which in turn should guide mitigating efforts more precisely.

Animal class	Species	Date	No. of animals	Description	Protected areas covering or adjoining the locality
Mammals	Wild Boar *Sus scrofa*	2019-09-25	12	Hit by a truck on a provincial road running through a coniferous forest, close to a forest clearing.	Protected area
	Wild Boar *Sus scrofa*	2015-07-23	4	–	Protected area
	Hedgehog *Erinaceus sp.*	2016-04-13	3	Ecotone of forest edge and wasteland.	Protected area
	Hare *Lepus europaeus*	2016-05-26	3	Wastelands and arable land; probably a female feeding her cubs on the road.	Protected area
	Red Fox *Vulpes vulpes*	2018-09-11	3	Forest edge and farmland.	Landscape park
	Moreover, PROS contains 11 further entries for 3 roadkilled mammals, 7 for hedgehogs and 4 for wild boars.				
Birds	Robin *Erithacus rubecula*	2016-04-10	105	Hel Peninsula on the Baltic Sea coast – a narrow (sometimes less than 200 m wide) wooded strip of land with a busy provincial road along. Important bird migration flyway.	SPA&SAC Natura 2000, IBA
	Brambling *Fringilla montifringilla*	2016-03-17	18	Flock hit by a truck (Kustusch & Wuczyński 2023).	
	Rook *Corvus frugilegus*	2016-05-22	8	Juveniles next to a nest colony (ca. 100 nests).	
	Tree Sparrow *Passer montanus*	2015-12-05	7	–	
	House martin *Delichon urbicum*	2017-05-21	7	By a pool of water. The birds were gathering mud for nest building.	
Amphibians	Common Toad *Bufo bufo*	2017-04-01	500	Ponds adjoining a residential area, arable land and grassland.	
	Unidentified	2018-04-06	500	–	SPA&SAC Natura 2000, IBA
	Common Toad *Bufo bufo*	2016-04-12	400	Spring migration to breeding ponds.	
	Common Frog	2019-03-17	250	Spring migration to breeding	Magurski National Park

	Rana temporaria			ponds.	(buffer zone), SAC Natura 2000
	Common Toad *Bufo bufo*	2017-04-01	240	Spring migration corridor by the river.	
Reptiles	Grass Snake *Natrix natrix*	2002-05-09	91	Forest interior in the Pilica river valley, close to the river and wetlands (Stolarz & Stolarz 2003).	SPA&SAC Natura 2000
	Grass Snake *Natrix natrix*	2019-05-27	60	Local road crossing a large fishpond complex and biodiversity hotspot (Borczyk 2004).	ornithological reserve "Milicz Ponds", landscape park, SPA&SAC Natura 2000, RAMSAR site, IBA, Living Lakes site
	Grass Snake *Natrix natrix*	2016-08-28	52	Basking on a road crossing large wetlands of international importance (Gryz & Krauze 2008, Hermaniuk & Ołdakowski 2016).	Biebrza National Park, SPA&SAC Natura 2000, RAMSAR site, IBA
	Grass Snake *Natrix natrix*	2010-09-25	20	Forest edge adjacent to the pond.	Protected area, SPA&SAC Natura 2000, IBA
	Grass Snake *Natrix natrix*	2018-09-02	20	Forest edge adjacent to the lake.	SPA&SAC Natura 2000, IBA

Explanations concerning the protected areas listed in the last column

Categories within the national system of protected areas in Poland

- National park – the highest category of protected area, of outstanding environmental, scientific, social, cultural and educational value,
- Nature reserve – high category of protected area, designated and managed for conservation purposes
- Landscape park – an area protected because of its natural, historical, cultural and scenic values
- Protected area – the least restrictive zones of protection in the Polish system taking into consideration manifold forms of outdoor recreation, e.g. hiking, cycling, canoeing, yachting, skiing and horse-riding

Categories within international systems of protected areas

- SPA Natura 2000 – Special Protection Area of the EU Natura 2000 network designated to safeguard the habitats of migratory and particularly threatened birds

- SAC Natura 2000 – Special Areas of Conservation of the EU Natura 2000 network designated to safeguard natural habitats and/or populations of the species other than birds
- RAMSAR site – a wetland site designated to be of international importance under the Ramsar Convention.
- IBA – BirdLife’s Important Bird and Biodiversity Area
- Living Lakes – an international network under the auspices of GNF, conserving fresh water resources, lakes and wetland ecosystems.

Figure S1

Correlation plot among observed roadkill of wildlife species in Poland, the species body mass, expected carcass persistence and distribution range in the country. Colours indicate positive (blue) or negative (red) correlation. The strength of the correlation is also highlighted by the proportion of the circle covered by each colour.

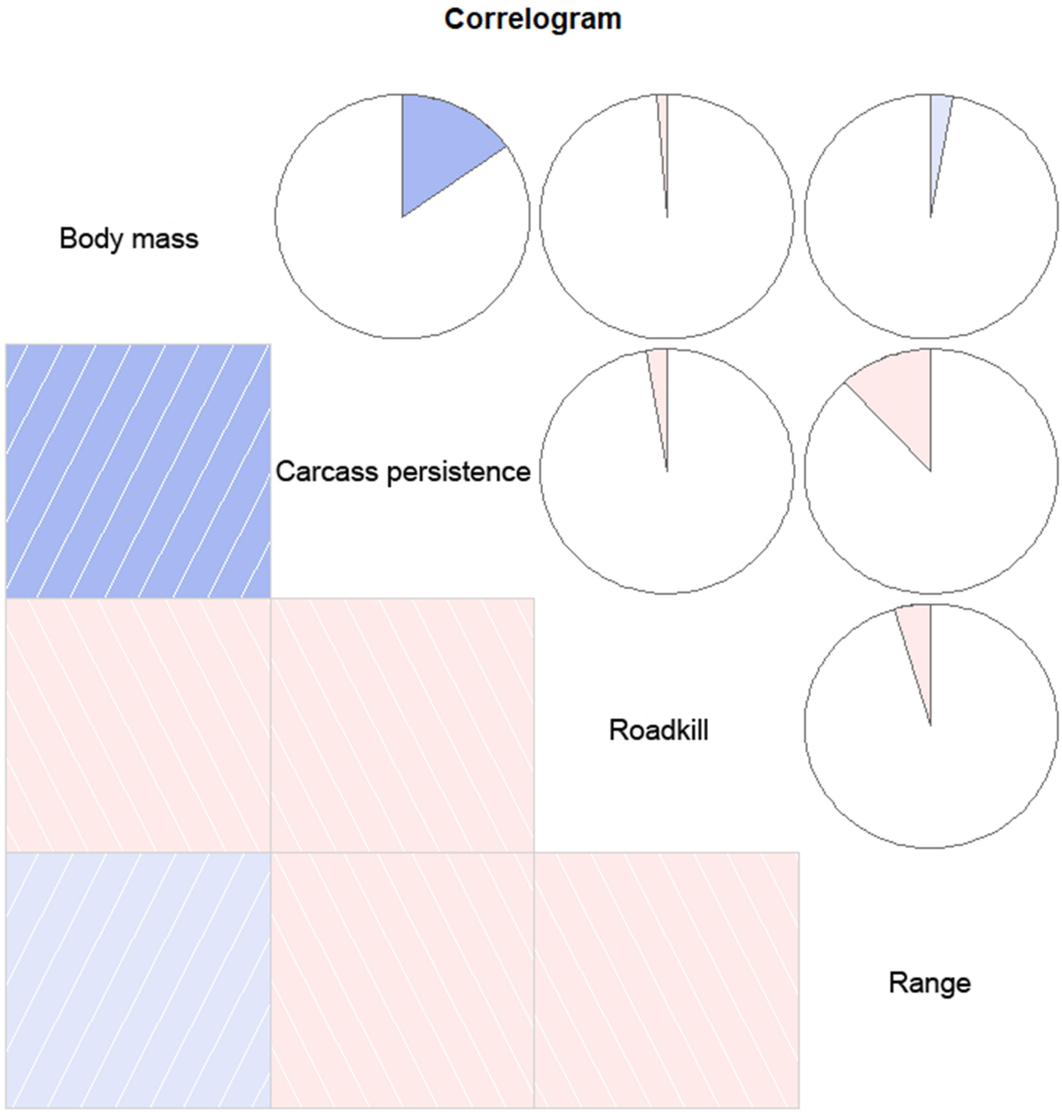

Figure S2

Roadkill records of selected animal species, collected in PROS until the end of November 2023.

Records of unidentified martens (497) and hedgehogs (4283) have been omitted.

PROS data reflect ecological characteristics of the species such as distribution and density differences in Poland, or seminatural processes like invasion events of alien species. Distribution of the two roadkilled marten species indicate their sympatric occurrence in the area of Poland (A). In contrast, roadkills of the two hedgehog species concentrate in opposite regions with likely contact zone somewhere in the mid-Poland, known from other studies (Wereszczuk & Zalewski 2015, Cerna Bolfikova et al. 2017) (B). Similarly, roadkill distribution of elk, European bison and hamster correspond to their range in the country (www.iop.krakow.pl/Ssaki) (C, D). Some other species as the white stork (E) and grey partridge (F) breed all over the Poland but their core populations and the highest densities are reported from eastern (Guziak & Jakubiec 2006, Wuczyński et al. 2021), and south-eastern part of the country, respectively (Kuczyński & Chylarecki 2012). Overall, the data confirm the relevance of information derived from citizen science projects in biodiversity monitoring at large spatial scales (van Strien et al. 2013, Callaghan et al. 2020, Petrovan et al. 2020).

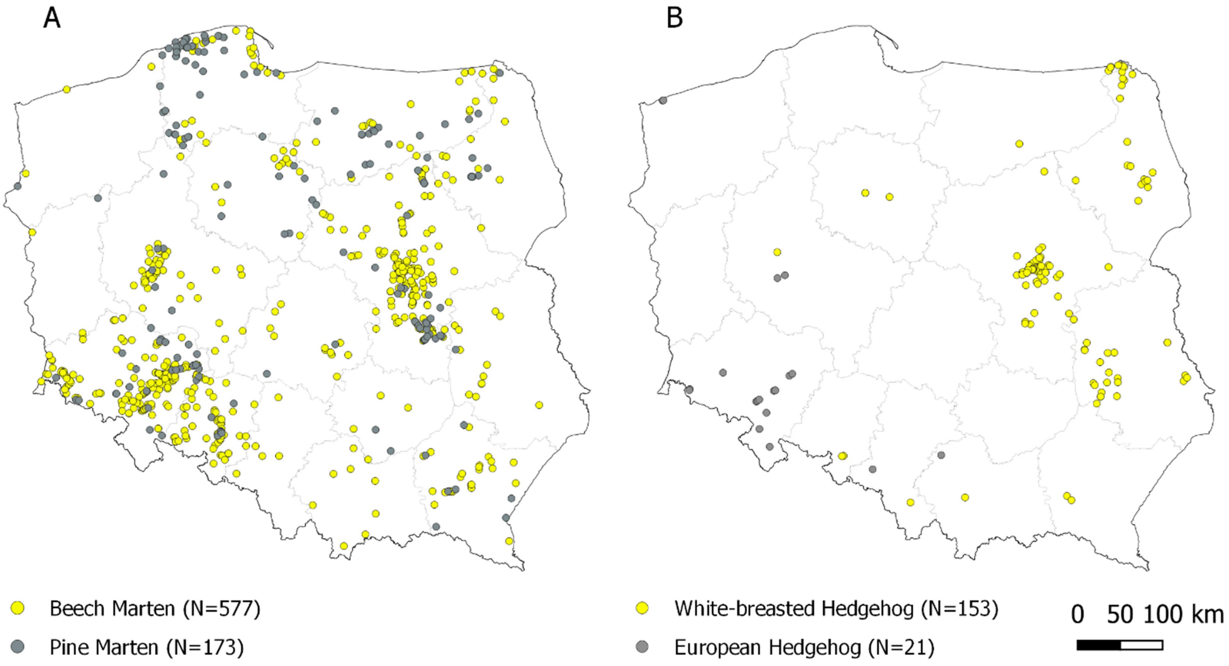

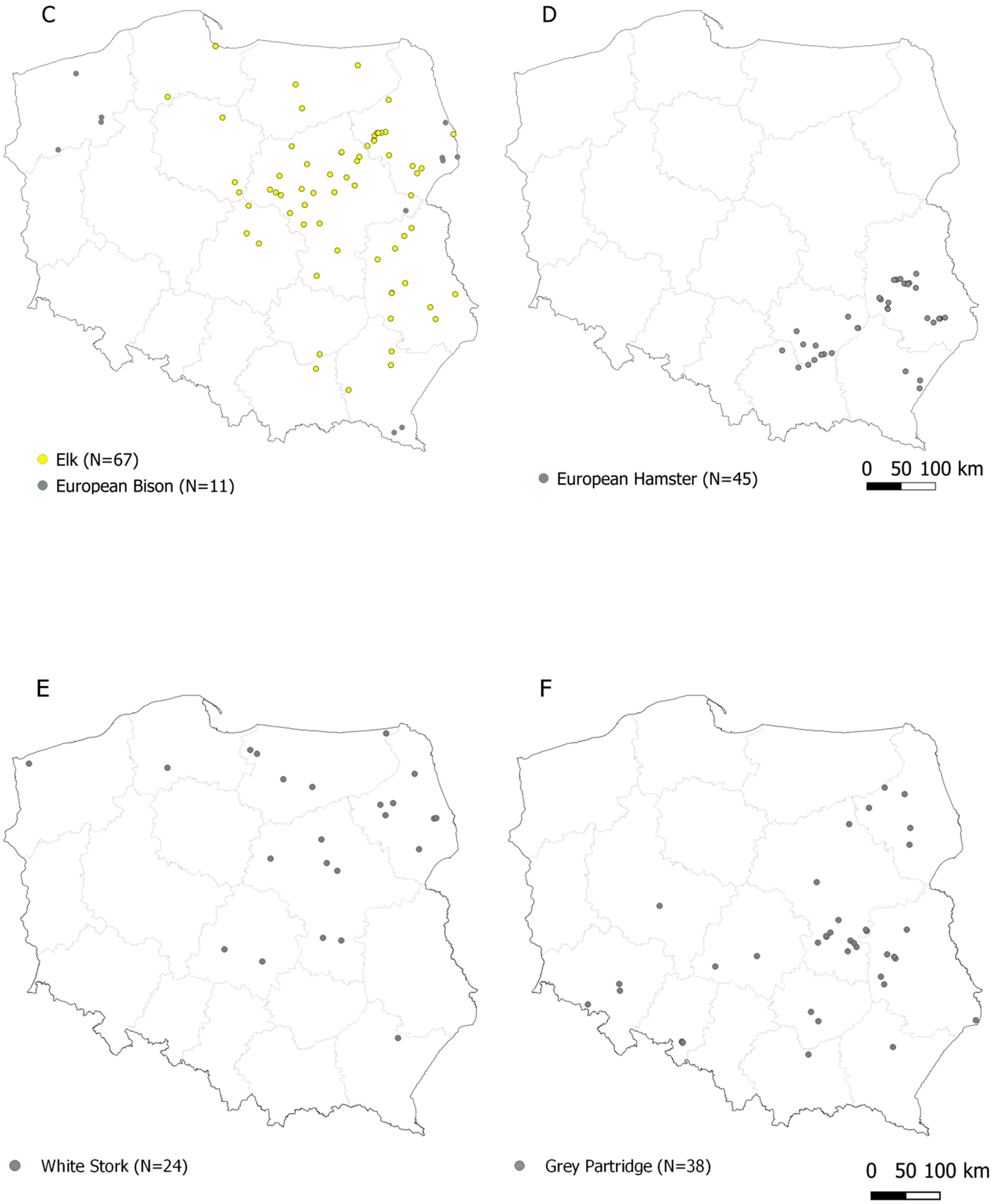
C
Elk (N=67)
European Bison (N=11)
D
European Hamster (N=45)
0 50 100 km
E
White Stork (N=24)
F
Grey Partridge (N=38)
0 50 100 km

References

Borczyk B. 2004. Causes of mortality and bodily injury in grass snakes (Natrix natrix) from the „Stawy Milickie" nature reserve (SW Poland). Herpetol Bull 90: 22-26.

Callaghan C. T., Roberts J. D., Poore A. G. B., Alford R. A., Cogger H., Rowley J. J. L. 2020. Citizen science data accurately predicts expert-derived species richness at a continental scale when sampling thresholds are met. Biodivers Conserv 29: 1323-1337.

Cerna Bolfikova B., Eliasova K., Loudova M., Krystufek B., Lymberakis P., Sandor A. D., Hulva P. 2017. Glacial allopatry vs. postglacial parapatry and peripatry: the case of hedgehogs. PeerJ 5: e3163.

Głowaciński Z. 2022. Red List of Polish vertebrates – updated version (1st and 2nd decade of the 21st century). Chronmy Przyr Ojczysta 78: 28-67.

Gryz J., Krauze D. 2008. Mortality of vertebrates on a road crossing the Biebrza Valley (NE Poland). Eur J Wildl Res 54: 709-714.

Guziak R., Jakubiec Z. 2006. Bocian biały Ciconia ciconia (L.) w Polsce w roku 2004. Wyniki VI Międzynarodowego Spisu Bociana Białego [White Stork Ciconia ciconia (L.) in Poland in 2004. Results of the VIth International White Stork Census]. PTPP „proNatura", Wrocław.

Hermaniuk A., Ołdakowski Ł. 2016. The mortality rate of vertebrates on the Tsar's Road in Biebrza National Park. Chronmy Przyr Ojczysta 72: 42-48.

Juszczyk W. 1987. Amphibians and reptiles of Poland. Państwowe Wydawnictwo Naukowe, Warszawa.

Kuczyński L., Chylarecki P. 2012. Atlas pospolitych ptaków lęgowych Polski. Rozmieszczenie, wybiórczość siedliskowa, trendy (Atlas of Common Breeding Birds in Poland: Distribution, Habitat Preferences and Population Trends). GIOŚ, Warszawa.

Kustusch K., Wuczyński A. 2023. Avian mortality on Polish roads – results of nationwide research based on citizen science. Ornis Polonica 64: 288-312.

Najbar A., Najbar B., Ogielska M. 2022. Selected aspects of biology, population structure and threats of the fire salamander Salamandra salamandra (L.) from Poland. Chronmy Przyr Ojczysta 78: 24-45.

Najbar B. 2006. The occurrence and the characteristics of Coronella austriaca austriaca (Laurenti, 1768)(Serpentes: Colubridae) in western Poland. Acta Zoologica Cracoviensia-Series A: Vertebrata 49: 33-40.

Petrovan S. O., Vale C. G., Sillero N. 2020. Using citizen science in road surveys for large-scale amphibian monitoring: are biased data representative for species distribution? Biodivers Conserv 29: 1767-1781.

Pucek Z. 1984. Key for identification of Polish mammals. Państwowe Wydawnictwo Naukowe, Warszawa.

Stolarz P., Stolarz E. 2003. Szlaki komunikacyjne – zagrozenie dla gadow w Dolinie Pilicy. Kraska 9: 37.

Storchová L., Hořák D. 2018. Life - history characteristics of European birds. Glob Ecol Biogeogr 27: 400-406.

van Strien A. J., van Swaay C. A. M., Termaat T. 2013. Opportunistic citizen science data of animal species produce reliable estimates of distribution trends if analysed with occupancy models. J Appl Ecol 50: 1450-1458.

Wereszczuk A., Zalewski A. 2015. Spatial Niche Segregation of Sympatric Stone Marten and Pine Marten – Avoidance of Competition or Selection of Optimal Habitat? PLoS ONE 10: e0139852.

Wilk T., Chodkiewicz T., Sikora A., Chylarecki P., Kuczyński L. 2020. Czerwona lista ptaków Polski. Ogólnopolskie Towarzystwo Ochrony Ptaków, Marki.

Wuczyński A., Krogulec G., Jakubiec Z., Profus P., Neubauer G. 2021. Population size and spatial distribution of the white stork *Ciconia ciconia* in Poland in 1958 with insights into long-term trends in regional and global population. The European Zoological Journal 88: 525-539.
